# Effect of nurse-led intradialytic stretching exercises on muscle cramp burden among patients undergoing maintenance hemodialysis: a randomized controlled trial

**DOI:** 10.1186/s12912-026-04430-4

**Published:** 2026-03-09

**Authors:** Ahmed Mostafa Shehata, Walaa Eid Zaki, Hamdya Ahmed Ali, Soheir Mohamed Weheida

**Affiliations:** 1 Medical Surgical Nursing Department, Faculty of Nursing, Beni-Suef University, Beni-Suef, Egypt; 2 Critical Care and Emergency Nursing Department, Faculty of Nursing, Beni-Suef University, Beni-Suef, Egypt; 3 Medical Surgical Nursing Department, Faculty of Nursing, Alexandria University, Alexandria, Egypt

**Keywords:** Hemodialysis, Muscle cramps, Intradialytic stretching, Nurse-led intervention, Randomized controlled trial, Symptom management, Egypt

## Abstract

**Background:**

Intradialytic muscle cramps disrupt hemodialysis (HD), trigger rescue interventions, and reduce patient comfort. Evidence for preventive, nurse-led strategies is limited. This study evaluated whether a standardized intradialytic stretching protocol reduces cramp burden compared to usual care.

**Methods:**

A single-center, parallel-group randomized controlled trial was conducted. Adults on maintenance HD with a history of leg cramps were randomized 1:1 to nurse-led stretching (*n* = 30) or usual care (*n* = 30). The intervention included supervised lower-limb stretches, twice weekly for ~ 8 weeks, with fidelity monitoring. Outcomes were assessed using the Arabic Muscle Cramp Severity and Characteristics Questionnaire. The primary outcome was post-intervention cramp intensity category.

**Results:**

Groups were comparable at baseline. Post-intervention, the study group showed significantly lower cramp intensity: no cramps in 83.3% vs. 30.0% of controls (*p* < 0.001). Cramp frequency, duration, pain, and discomfort all favored the intervention (all *p* < 0.01). Leg temperature perception did not differ. No serious adverse events occurred.

**Conclusions:**

A brief, nurse-led stretching protocol produced large reductions in cramp burden without disrupting workflow. Integrating standardized stretching into routine nursing care is a feasible, low-cost strategy for proactive symptom management, empowering nurses and enhancing patient comfort. Multicenter, longer-term evaluations are warranted.

**Clinical trials:**

The trial is registered at ClinicalTrials.gov under identifier NCT07262879.

**Registration date:**

12/02/2025.

**Supplementary Information:**

The online version contains supplementary material available at 10.1186/s12912-026-04430-4.

## Introduction

Muscle cramps during hemodialysis (HD) are common and troubling complications. They strongly affect patient comfort, adherence to treatment, and overall quality of life. These painful, involuntary muscle contractions most often occur in the calves and feet, but they can also involve other muscles in the lower limbs. Estimates indicate that 20 to 85% of patients on chronic HD experience cramps, with significant variation due to differences in patient demographics, dialysis protocols, and definitions of cramp episodes [1, 2]. From a nursing perspective, cramps that occur during dialysis require immediate actions such as massage, saline boluses, or adjustments in dialysis settings. These interventions can disrupt workflows, increase workload, and possibly expose patients to issues such as changes in blood pressure [3].

Nursing professionals are crucial in identifying, managing, and preventing muscle cramps [4]. Their presence at the bedside during HD sessions allows them to assess early signs in real time. Signs may include discomfort, restlessness, or patient-initiated alarms. This enables quick implementation of preventive measures [5]. However, existing interventions have been primarily reactive, aiming at relief rather than prevention. This highlights the need for affordable, nurse-led strategies that can easily fit into routine HD care [6, 7].

Intradialytic stretching exercises are non-pharmacological, non-invasive interventions that fall within the nursing scope of practice, as they optimize patient health, prevent complications, and promote functional abilities when performed competently and safely by trained nurses [8, 9]. Stretching is believed to improve muscle flexibility, increase local blood flow, and manage neuromuscular excitability, which may reduce the number of triggers that cause cramps [10]. Nurse-led stretching protocols can use time during HD sessions to offer a structured, patient-focused preventive strategy. This approach aligns with nursing values such as advocacy, continuity of care, and health promotion [11].

Emerging but limited evidence supports the effectiveness of stretching during HD. For example, small pilot studies have shown fewer cramp occurrences and lower pain scores after lower limb stretching routines conducted during HD sessions [12]. Another quasi-experimental controlled study reported a significant drop in cramp severity and frequency in the intervention group post-stretching compared with control [13]. Despite these encouraging results, many studies lack rigorous methods, standardized protocols, or sufficient participant numbers, which prevents widespread adoption in nursing practice [14]. In the Egyptian context, while HD nursing care is comprehensive, standardized, protocol-driven intradialytic stretching exercises are not yet a routine part of clinical practice, representing a significant gap in proactive symptom management [15].

From a nursing management viewpoint, a structured stretching protocol has several potential benefits. This may lessen the need for reactive measures such as saline infusion or changing the ultrafiltration rate, reducing the risk of blood pressure drop or fluid overload [16]. Additionally, integrating stretching into dialysis sessions encourages patient involvement and improves the nurse–patient relationship, building trust and promoting patient empowerment in self-management [17].

However, introducing intradialytic stretching into nursing practice faces practical and educational barriers. Nurses need training to perform standardized stretches correctly, monitor how patients respond, and adjust techniques on the basis of mobility issues or access sites. Institutional support, which includes proper staffing, time management, and documentation processes, is essential. Without this support, even effective interventions may struggle in real-world settings [18].

In the HD unit at Beni-Suef University Hospital, nursing staff and patients have reported cramps as a frequent source of session interruption and distress, particularly among older patients or those with diabetes, low blood pressure, or long-term dialysis [15]. These findings reflect global trends. In response, our nursing team launched a quality improvement plan to test an intradialytic stretching protocol designed to suit the local environment, focusing on low-resource feasibility, nurse autonomy, and patient safety.

This study aimed to evaluate whether a standardized intradialytic stretching protocol reduces cramp burden compared to usual care. Using a parallel-group RCT design (as outlined in the Methods), we gathered valid data to inform evolving nursing standards. We believed that, compared with standard care, the stretching intervention significantly reduced the incidence, duration, and intensity of cramps by the end of the study (8 weeks). We also expected that both nurses and patients would accept the intervention well, providing a practical model for proactive cramp management in HD settings.

## Study design

This study used a single-center, parallel-group randomized controlled trial design to assess the effect of a nurse-led intradialytic stretching program on muscle cramps in adults receiving maintenance hemodialysis (HD).

### CONSORT guidelines

This study was conducted and reported in accordance with the Consolidated Standards of Reporting Trials (CONSORT) 2025 guidelines.

### Setting

The study took place in the HD unit of Beni-Suef University Hospital, a teaching hospital in Egypt. The unit serves adult patients on three-weekly HD (approximately 4 h per session), operates multiple shifts each day, and has 25 dialysis stations, including dedicated stations for patients with viral hepatitis. The nurse-to-patient ratio is maintained at 1:4, ensuring continuous observation, medication administration, and technical monitoring. Routine care, which includes ultrafiltration targets, dialysate composition, blood flow rates, and blood pressure monitoring, is directed by physicians and delivered by nurses. The unit uses a hybrid system of electronic and paper records to collect demographic, clinical, and dialysis-related data.

### Sample and sampling

The sample size was pragmatically determined based on feasibility and patient flow within the study timeframe, aiming to detect clinically meaningful differences. The sampling frame comprised all adult patients (≥ 21 years) on maintenance HD at the study site during the recruitment period. The inclusion criteria were as follows: (1) received three-weekly HD for ≥ 6 months; (2) were clinically stable; and (3) had a history of intradialytic lower-limb muscle cramps in the preceding month. The exclusion criteria were as follows: (1) musculoskeletal or neurological disorders limiting safe lower-limb stretching; (2) femoral vascular access or recent lower-limb vascular procedures; and (3) first or emergency HD sessions. Consecutive eligible patients were approached by trained HD nurses. Those who provided informed consent were enrolled until the target sample was achieved (*n* = 60; 30 per arm). Randomization was performed with computer-generated permuted blocks (size = 4), with stratification by dialysis shift (morning/afternoon) and baseline cramp frequency (≥ 3 vs. < 3 episodes per session) to promote balance of prognostically relevant factors.

### Data collection tools

#### Muscle Cramp Severity and Characteristics Questionnaire (MC-SCQ, Arabic version)

The Muscle Cramp Severity and Characteristics Questionnaire (MC-SCQ) is a five-item instrument originally developed in English by Morris (2014) to assess intradialytic muscle cramp burden [19]. For this study, we created and validated an Arabic version to ensure cultural and linguistic appropriateness for our patient population.

##### Adaptation and validation of the Arabic MC-SCQ

The adaptation process followed established cross-cultural translation guidelines. The English MC-SCQ was forward-translated into Arabic by a bilingual clinical nurse specialist, then independently back-translated by a bilingual methodologist, with discrepancies resolved by consensus. Cognitive interviews with hemodialysis patients were conducted using think-aloud and probing techniques, leading to lexical refinements (e.g., using colloquial terms for “cramp”). The prefinal version was reviewed by an expert panel of nephrology nurses and a consultant nephrologist in a three-round process to establish content validity.

Psychometric evaluation of the Arabic MC-SCQ demonstrated: satisfactory internal consistency (Cronbach’s alpha); good test-retest reliability over 7–10 days in stable patients (intraclass correlation coefficients); and evidence of convergent validity through positive correlations with an 11-point numeric pain rating scale. Pilot testing confirmed the tool’s clarity, appropriate score distribution without floor/celling effects, and feasibility for clinical administration.

The Arabic MC-SCQ assesses five domains over a two-week recall period: (a) cramp frequency per hour, (b) average duration in minutes, (c) pain intensity, (d) perceived leg temperature change (cool/neutral/warm), and (e) discomfort/functional interference. Four items (frequency, duration, pain, discomfort) are scored 0–3 using anchored ordinal scales; temperature contributes 0–1 point. Total scores (0–13) categorize cramp intensity as: none (0), mild (1–4), moderate (5–8), or severe (9–13). The adapted Arabic version was used in this study.

#### Intervention adherence and fidelity checklist

An observational tool, the Intervention Adherence and Fidelity Checklist, was developed by the research team to standardize and quantify the delivery of the intradialytic stretching protocol. Its purpose is to ensure procedural consistency, treatment fidelity, and reproducible intervention efficacy across all sessions. The checklist operationalizes four core stretching components: ankle dorsiflexion, gastrocnemius stretching, soleus stretching, and hamstring stretching. Each component is evaluated across six domains: correct patient positioning, adherence to prescribed stretch duration (20–30 s), completion of the stipulated repetitions (three per limb), maintenance of vascular access safety, monitoring of patient discomfort, and general safety. Performance on each domain is scored using a three-point criterion (0 = incorrect/not performed; 1 = partially correct; 2 = fully correct), yielding a total fidelity score ranging from 0 to 40, with higher scores indicating greater adherence.

Content validity was established via expert panel review (comprising nephrology nurses, a physiotherapist, and a nephrologist), who assessed item relevance, clarity, and comprehensiveness. Inter-rater reliability was determined through concurrent, independent observation of a purposive sample of sessions by two trained nurse assessors. Agreement was quantified using Cohen’s kappa for individual items and the intraclass correlation coefficient for total scores. Discrepancies were resolved through discussion to refine scoring criteria. The instrument was developed in Arabic to align with clinical practice, with English translations provided for training purposes.

#### Case Report Form (CRF)

A structured Case Report Form (CRF) was designed to systematically capture patient characteristics and dialysis-related variables. Its function is to standardize data collection, ensuring accuracy and reliability. The CRF includes predefined variables with operational definitions to guide abstraction. These encompass patient demographics (age, gender, marital status, education), clinical comorbidities (e.g., diabetes mellitus, peripheral vascular disease), dialysis vintage, and prescription details (blood flow rate, dialyzer specifications, session duration, ultrafiltration rate). It also records interdialytic weight gain and pre- and post-dialysis blood pressures.

Validity was assessed through review by senior nephrology nursing leadership. Reliability was evaluated via inter-abstractor agreement, whereby two independently trained abstractors completed the CRF for a 10% sample of patient records. High agreement was observed across variables; any discrepancies informed clarifications to the form’s definitions and instructions, thereby enhancing the accuracy and quality of subsequent data collection.

### Data collection and intervention procedure

Screening and baseline assessments were conducted by trained nurses during routine hemodialysis sessions. Following informed consent, participants completed the baseline CRF and the Arabic version of the MC-SCQ. An independent coordinator then performed randomization using sequentially numbered, opaque, sealed envelopes.

The intervention consisted of a supervised, standardized lower-limb stretching protocol administered by HD nurses during the first 1–2 h of dialysis. The protocol was developed based on principles of static stretching and neuromuscular physiology, targeting the major muscle groups prone to cramping. It included: (1) ankle dorsiflexion, (2) passive gastrocnemius stretch, (3) passive soleus stretch, and (4) hamstring stretch. Each stretch was held for 20–30 s and repeated three times per limb, with nurses providing verbal encouragement and monitoring for discomfort or access-site compromise. The intervention was delivered twice weekly (rather than during every session) based on a balance between feasibility, potential benefit, and avoiding patient fatigue or interference with other aspects of dialysis care. Adherence was documented using the fidelity checklist after each session. Control group participants received usual care without formal stretching. Adverse events (e.g., hypotension, dizziness, cramping) were monitored throughout, with predefined criteria for procedure discontinuation. Post-intervention outcome assessment (MC-SCQ) was conducted by assessors blinded to group allocation wherever possible, with procedures adjusted to maintain blinding.

#### Nurse training

Prior to participant enrollment, nurses underwent competency-based training covering relevant anatomy and biomechanics, vascular access safety (emphasizing avoidance of the access limb), correct execution of the four stretches, patient communication, and documentation protocols. Nurses were assessed for competency before being authorized to deliver the intervention.

### Ethical considerations

The study was approved by the institutional Research Ethics Committee prior to initiation; the protocol adhered to the ethical principles of the Declaration of Helsinki and local regulatory requirements. All participants provided informed consent after a nurse explained the study aims, procedures, potential benefits, and risks in accessible language. Participation was voluntary, and patients could withdraw at any time without affecting their care. Privacy and confidentiality were protected through coded identifiers, secure storage, and restricted access to linkage files. The intervention was designed for safety in the HD context: stretches were delivered away from the vascular access limb when necessary, with continuous monitoring of blood pressure and symptoms; any hypotension, severe pain, or access instability triggered immediate cessation and clinical management. No additional cost was borne by the participants. The study planned prompt reporting of adverse events to the ethics committee and the unit’s clinical governance lead. Because this was a single-center trial of a low-risk behavioral intervention embedded in standard care, formal data safety monitoring was performed by the clinical governance team with periodic review of recruitment, adherence, and safety logs.

#### Use of artificial intelligence (AI)

During manuscript preparation, the authors used DeepSeek (an AI language model) solely to assist with paraphrasing and improving textual clarity in select sections. The AI tool did not contribute to the study design, data collection, analysis, interpretation, or scientific insight generation. All intellectual content remains the original work of the authors, who take full responsibility for the final manuscript.

## Results

A total of 60 patients were randomized (study = 30; control = 30). The groups were well balanced at baseline across sociodemographic characteristics, comorbidities, and key hemodynamic/laboratory parameters. Postintervention comparisons consistently favored the stretching group across multiple cramp-related outcomes (side, muscle involved, frequency, duration, pain, and discomfort), whereas leg temperature did not differ. Pre–post distributions of cramp intensity showed marked improvement in the study group but minimal change in the control group. The age structure was broadly comparable, with most patients clustered between 30 and 59 years. Although the study arm had a somewhat greater proportion in the 30–39 band (33.3% vs. 20.0%) and fewer in the 50–59 band (20.0% vs. 40.0%), the omnibus chi-square for age categories indicated no statistically significant imbalance (χ² = 3.392; *p* = 0.494). Gender, marital status, occupation, education, and residence were also similar (all *p* > 0.05) (Table 1).

**Table 1:** Demographic characteristics

Patients’ characteristics	Study Group (*n* = 30)	Control Group (*n* = 30)	χ²	*P* value
**Age (years)**			**3.392**	**0.494**
21–29	2 (6.7%)	1 (3.3%)		
30–39	10 (33.3%)	6 (20.0%)		
40–49	9 (30.0%)	8 (26.7%)		
50–59	6 (20.0%)	12 (40.0%)		
≥ 60	3 (10.0%)	3 (10.0%)		
**Gender**			**0.601**	**0.438**
Male	17 (56.7%)	14 (46.7%)		
Female	13 (43.3%)	16 (53.3%)		
**Marital status**			**0.800**	**0.371**
Married	21 (70.0%)	24 (80.0%)		
Unmarried	9 (30.0%)	6 (20.0%)		
**Occupation**			**1.952**	**0.582**
Not working	8 (26.7%)	10 (33.3%)		
Worker	6 (20.0%)	7 (23.3%)		
Retired	7 (23.3%)	3 (10.0%)		
Free business	9 (30.0%)	10 (33.3%)		
**Education**			**0.332**	**0.954**
Illiterate	10 (33.3%)	12 (40.0%)		
Read & write	9 (30.0%)	8 (26.7%)		
Secondary	6 (20.0%)	5 (16.7%)		
University	5 (16.7%)	5 (16.7%)		
**Residence**			**0.268**	**0.605**
Urban	15 (50.0%)	17 (56.7%)		
Rural	15 (50.0%)	13 (43.3%)		

As shown in Table 2, the comorbidity profiles were similar. Cardiovascular disorders were common in both groups (53.3% vs. 50.0%), with comparable frequencies of endocrine, hematological, respiratory, neurological, and immunological conditions (all *p* > 0.39). The absence of significant between-group differences indicates that downstream differences in cramp outcomes are unlikely to be explained by baseline multimorbidity.

**Table 2:** Clinical comorbidities

Comorbidities (+)	Study *n* (%)	Control *n* (%)	χ²	*P* value
Endocrine	11 (36.7)	13 (43.3)	0.278	0.598
Hematological	6 (20.0)	8 (26.7)	0.373	0.542
Cardiovascular	16 (53.3)	15 (50.0)	0.067	0.796
Respiratory	6 (20.0)	7 (23.3)	0.098	0.754
Neurological	10 (33.3)	7 (23.3)	0.739	0.390
Immunological	11 (36.7)	12 (40.0)	0.071	0.791
Others	6 (20.0)	5 (16.7)	0.111	0.739

Additionally, Table 3 shows that the mean calcium level, interdialytic weight per session, and blood pressure were nearly identical across arms (all *p* ≥ 0.58). The tight alignment of these physiological markers further supports baseline equivalence and reduces the risk that subsequent cramp differences reflect underlying hemodynamic divergence.

**Table 3:** Baseline hemodynamic and laboratory parameters

Variable	Study (Mean ± SD)	Control (Mean ± SD)	t value	*P* value
Calcium level	8.52 ± 1.30	8.65 ± 1.43	−0.349	0.728
Weight/session (kg)	73.40 ± 12.95	75.17 ± 12.09	−0.546	0.587
Systolic BP (mmHg)	136.67 ± 21.54	136.33 ± 17.71	0.065	0.948
Diastolic BP (mmHg)	84.67 ± 12.24	84.00 ± 13.54	0.200	0.842

After the intervention period (Table 4), the absence of cramps was far more common in the stretching arm: 83.3% reported no side involvement, whereas 30.0% reported no side involvement in the controls (absolute difference = 53.3 pp; χ² = 17.529; *p* < 0.001). When cramps did occur, they were predominantly unilateral in controls (right 36.7%, left 33.3%). In terms of musculature, “no muscle involved” (i.e., no cramps) reached 83.3% in the study group and 30.0% in the control group (χ² = 17.537; *p* = 0.001), whereas calf, hamstring, and soleus cramps clustered in the control arm. These patterns are consistent with the clinically meaningful protective effect of the stretching protocol.

**Table 4:** Side of cramp and muscle involved (postintervention)

Outcome	Study *n* (%)	Control *n* (%)	χ²	*P* value
**Side of muscle cramps**			**17.529**	**0.000**
No	25 (83.3)	9 (30.0)		
Right	3 (10.0)	11 (36.7)		
Left	2 (6.7)	10 (33.3)		
**Muscle involved (+)**			**17.537**	**0.001**
No	25 (83.3)	9 (30.0)		
Calf	3 (10.0)	10 (33.3)		
Hamstring	1 (3.3)	6 (20.0)		
Soleus	1 (3.3)	5 (16.7)		

As shown in Table 5, across multiple dimensions, the stretching group showed substantial improvement. Frequency: “not occurring” was observed in 83.3% vs. 30.0% (χ² = 19.085; *p* < 0.001) of the participants; high-frequency episodes (> 3/hour) occurred only in the controls (26.7%). Duration: Nonoccurrence again reached 83.3% vs. 30.0% (χ² = 18.471; *p* < 0.001), whereas protracted cramps (> 5 min) were common in controls (50.0%). Pain: A total of 83.3% of the study group reported no pain, whereas 30.0% of the controls reported severe pain, with severe pain evident in 26.7% of the controls and none in the study arm (χ² = 26.637; *p* < 0.001). There was no difference in leg temperature between groups (*p* = 0.785), suggesting that the intervention influenced neuromuscular/functional dimensions more than did perceived thermal changes. Discomfort/quality: Most study patients reported no discomfort (83.3% vs. 30.0%), whereas “painful” or “unbearable” levels were clustered in the control group (χ² = 18.463; *p* = 0.001). Collectively, these findings indicate a broad, consistent reduction in cramp burden attributable to the nurse-led stretching program.

**Table 5:** Detailed cramp characteristics after the intervention

Domain	Category	Study *n* (%)	Control *n* (%)	χ²	*P* value
Frequency	Not occur	25 (83.3)	9 (30.0)	19.085	0.000**
	< 3 times/hour	5 (16.7)	13 (43.3)		
	> 3 times/hour	0 (0.0)	8 (26.7)		
Duration	Not occur	25 (83.3)	9 (30.0)	18.471	0.000**
	< 5 min	3 (10.0)	6 (20.0)		
	> 5 min	2 (6.7)	15 (50.0)		
Level of pain	No pain	25 (83.3)	9 (30.0)	26.637	0.000**
	Mild	4 (13.3)	1 (3.3)		
	Moderate	1 (3.3)	12 (40.0)		
	Severe	0 (0.0)	8 (26.7)		
Leg temperature	Warm	23 (76.6)	25 (83.3)	0.483	0.785
	Cold	6 (20.0)	4 (13.3)		
	Clammy	1 (3.3)	1 (3.3)		
Discomfort/quality	No	25 (83.3)	9 (30.0)	18.463	0.001**
	Perceptible	2 (6.7)	4 (13.3)		
	Sensitive	1 (3.3)	5 (16.7)		
	Painful	2 (6.7)	8 (26.7)		
	Unbearable	0 (0.0)	4 (13.3)		

Note: (**) Highly significant at *p* < 0.001

Preintervention, the intensity distributions were comparable (χ² = 1.168; *p* = 0.761). Postintervention, the study group shifted dramatically toward “no cramps” (83.3%) with a near elimination of moderate–severe categories (6.6% combined), whereas the control group showed little improvement (30.0% “no cramps,” moderate–severe remaining 60.0%). The between-group difference at postintervention was large and highly significant (χ² = 20.409; *p* < 0.001), which is consistent with a robust intervention effect (Table 6).

**Table 6:** Distribution of cramp intensity before and after the study vs. control

Intensity	Study Pre *n* (%)	Control Pre *n* (%)	Study Post *n* (%)	Control Post *n* (%)
No cramps	7 (23.3)	8 (26.7)	25 (83.3)	9 (30.0)
Mild	6 (20.0)	4 (13.3)	3 (10.0)	3 (10.0)
Moderate	15 (50.0)	14 (46.7)	1 (3.3)	12 (40.0)
Severe	2 (6.7)	4 (13.3)	1 (3.3)	6 (20.0)
**χ² (P value)**	**—**	**Pre: 1.168 (0.761)**	**—**	**Post: 20.409 (0.000**)**

## Discussion

This randomized study clearly demonstrates that a simple protocolized stretching procedure performed by dialysis nurses is effective in ameliorating the intradialytic muscle cramping phenomenon in various aspects of frequency, intensity, duration, laterality, and discomfort, but not affecting the subjective feelings of leg temperatures. Because the effects were found to be internally consistent across various endpoints, it suggests an authentic treatment effect, not just an artifact of measurement error. Also, it validates in the expected direction, based on its underlying scientific principles of neuromuscular changes due to stretching interventions [20]. Also, by adding a fidelity measure to a simple protocolized stretch activity performed during standard patient care, the study is an advancement from the previous literature, in which instruction was often vague regarding home exercise programs [21].

The degree of improvement is clinically relevant. The degree of improvement from the moderate to severe categories to “no cramps” or “mild” in the intervention group was large in comparison to the negligible changes in the control group. The degree of improvement in the intervention group would indicate the degree to which the cramping has been alleviated systematically during dialysis. Cramps are the most disturbing sort of intradialytic symptoms that lead to alarms, nurse calls, and changes in ultrafiltration rates or dialysis parameters. As such, their alleviation could improve the continuity of the procedure and the patient comfort, which fit the interests of the nursing staff [22]. For the discipline of symptom science, the degree of improvement, in terms of fewer episodes, shorter episodes, and reduced pain, represents aspects that by themselves provide converging validity to the observations.

Biologically plausibility enhances validity. Static stretching acutely increases muscle-tendon stiffness, activates inhibitory spinal circuits (e.g., autogenic inhibition mediated by the golgi tendon organs), and decreases muscle spindle sensitivity, reducing alpha motor neuron excitability—a basis for spasm generation [23]. Conditions associated with dialysis, potentially mitigating the threshold values of spasm generation: fluid shifts, Na/K, Ca adjustments, sympathetic variability during the procedure: these may be offset, at least in part, by the neuromechanical adjustments [24]. Differences among the groups are not unexpected in the ratings of leg temperature, being consistent with the neuromuscular mechanism: the temperature ratings are more closely associated with blood flow in the skin than alpha-innervation excitability, active spasm not being expected to affect the former [25]. This suggests additional specific information than an inability to alleviate spasm.

Methodological aspects enhance credibility. Random allocation with allocation concealment ensured that there was little opportunity for selective bias in subject allocation, and initial sociodemographic and physiological differences were made equal, with prespecified outcomes subjected to traditional analysis strategies suited to their nature. Crucially, however, intervention implementation involved clear holds and repetitions (20–30 s, three cycles on each limb) targeting dorsiflexors and gastrocnemius-soleus hamstrings, that is, exactly those muscle groups most frequently involved in intradialytic bouts. The session-level fidelity assessment measured intervention implementation regarding positioning, duration, cycle number, and vascular access integrity. The thrust here, clearly, is attention to intervention implementation, in which, as pointed out in a different area of physical rehabilitation, fidelity is usually merely assumed rather than formally quantified and provides a template for replication on a broader scale [26].

The approach to measurement also warrants comment. The Arabic Muscles Cramp Severity and Characteristics Questionnaire (MC-SCQ) assessed burden on scales of five dimensions – frequency, duration, pain, leg temperature perception, and comfort or functional interference – summed to an interpretable score ranging from 0 to 13 by category. Content validity and test–retest validity from pilot testing offer assurance of suitability for the dialysis setting, where transparency and cultural appropriateness are key priorities. Crucially, use of multiple dimensions mitigates the potential for benefit appearing to be indicated by a single “unstable” item – although congruent change on the dimensions of frequency, duration, pain, and comfort, with a non-significant result for temperature, indicates responsive change consistent with the mechanism of the intervention.

The result is not merely evidencing a persuasive pattern, as it is also clinically interpretable. Severe pain had all but vanished in the stretching group, though it remained present in a notable fraction of controls, while high-frequency and prolonged cramps, presumably causing the most functional impairment, occurred predominantly among controls. Bilateral distributions introduce a further complication: the preponderance of unilateral cramps among controls, combined with a strong “no side” group among cramps in the treatment group, suggest a reduced number of events and a reduced spread distribution of cramp-susceptible muscle groups. In sum, a pattern of general cramp-susceptibility reduction rather than mere subjective pain reduction is evident.

To situate the findings of the present work among those that have come before, these findings support the increasingly clear literature that intradialytic exercise protocols, particularly those focusing on flexibility and activation in the lower limbs, may decrease symptom intensity and enhance tolerability of treatment [27]. The present work adds to that literature in that it (i) delivered the stretching protocol in a tightly standardized manner, (ii) administered the treatment protocol within the framework of nursing practice protocols with competency-based training, and (iii) assessed treatment fidelity simultaneously with patient care. Such a triangulation, ranging from the clarity of the treatment protocol to the challenges of working within clinical practice, may provide the tools for achieving the significant treatment effects often absent when successful treatment protocols ‘fail to survive’ the transition to a busy clinical practice setting [28].

There are other explanations that should be borne in mind. Regression towards the mean would have predicted similar improvements for the groups, as both started from a similar level, but instead, the control group performance remained stagnant, while the intervention group performance changed dramatically, making this explanation implausible. The expectancy effect would lead to an improvement in the patients’ reported level of pain and discomfort, but the fact that the changes have scale and consistency, coupled with the change being categorical and frequency or duration-based, makes it hard to put down to mere perception. Contamination is a risk for nurse-delivered therapies, but the significant separation of the groups makes it implausible as an explanation for the result. The prescription of dialysis and the increases in weight during the periods between dialysis sessions, both potential sources of contamination, were similar at the start, and the pattern of the intervention effect would suggest a specific rather than general change to the dialysis environment [29].

Taken together, the data support a clear conclusion: a brief, nurse-implemented stretching program, accomplished with good fidelity, could reduce the complex distal effect of intradialytic muscle cramping. The consistency of the outcomes, biological plausibility, and attention to implementation quality combined allow the scientific community to move from concepts of promise to intervention [30].

### Implications for nursing practice and service delivery

In brief, a protocol involving a lower-limb stretching sequence during the first 60–90 min of hemodialysis reframes cramp care from reactive rescue to proactive preventive nursing practice. Because a routine requires no specialized equipment, it can be embedded within chairside workflows and aligned with existing monitoring tasks. A concise visual protocol paired with a short fidelity checklist (covering positioning, hold time, repetitions, vascular access safety, and patient comfort) supports competency development, standardization, and audit/feedback while also reinforcing a safety culture around the access limb. Guided stretching further cultivates patient engagement and self-efficacy, as nurses coach breathing, alignment, and protected handling of the access arm, translating abstract advice into reproducible skills patients can continue between sessions. By lowering the frequency, duration, and pain of cramps, the approach is also likely to reduce alarms, unplanned saline boluses, and ultrafiltration adjustments—improvements that can enhance session continuity and allocate nurse time more efficiently, even though these operational gains warrant formal measurement.

## Limitations

Interpretation should consider several constraints. The study was conducted at a single tertiary unit with a modest sample, which may limit its generalizability to settings with different staffing models, dialysis prescriptions, or patient case mixes. Blinding of participants and treating nurses was not feasible, so expectancy and performance effects remain possible despite prespecified outcomes and fidelity monitoring. Follow-up was limited to approximately eight weeks, leaving the durability of benefits and any carry-over between sessions uncertain. Although the Arabic MC-SCQ has demonstrated local content validity and temporal stability, it has not yet been externally validated against physiological indices (e.g., surface electromyography) or ecological momentary sampling. Furthermore, outcome assessment was conducted at the end of the 8-week period but not immediately post-dialysis, which could have provided additional insights into the acute effects of stretching within a single session. Finally, process outcomes such as rescue saline use, ultrafiltration changes, intradialytic hypotension, and time‒motion impacts were not systematically captured, and informal stretching in controls or variability in adherence dose may have occurred despite the pronounced between-group separation.

## Future directions

Future work should test scalability and sustainability through pragmatic multicenter trials—ideally cluster-randomized or stepped-wedge designs—with longer follow-up periods to examine durability, quality of life, and health-care utilization. Mechanistic studies that pair the protocol with electrophysiology and microcirculatory monitoring could delineate neuromuscular pathways, refine dosing parameters (hold durations, repetitions, session frequency), and tailor the intervention to specific cramp phenotypes. Implementation science frameworks (e.g., the Consolidated Framework for Implementation Research (CFIR)) can identify adoption barriers and facilitators, optimize training, and integrate fidelity tracking into electronic dialysis flowsheets. Economic evaluations are also needed to relate training and delivery costs to potential reductions in alarms, interruptions, and acute interventions. Finally, research should explore personalization by age, diabetes, peripheral vascular disease, dialysis vintage, and access type; adapt the protocol for mobility limitations; and assess hybrid strategies or digital support (e.g., brief video prompts) to sustain adherence over time.

## Conclusion

A concise, nurse-led stretching protocol integrated into routine hemodialysis sessions produced large, coherent reductions in intradialytic cramp burden across frequency, duration, pain, and discomfort without compromising access safety or workflow. The intervention is feasible, teachable, and compatible with real-world practice, providing a practical pathway to embed preventive, patient-centered symptom management within dialysis care while broader validation and longer-term evaluation proceed.

## Supplementary Information

Below is the link to the electronic supplementary material.

Supplementary Material 1

Supplementary Material 2

## Data Availability

The datasets generated and/or analyzed during the current study are available from the corresponding author upon reasonable request. The study instruments used in the data collection (Arabic MC-SCQ and the Intervention Adherence & Fidelity Checklist) are available upon request for academic use.

## Abbreviations

CFIR: Consolidated Framework for Implementation Research
CONSORT: Consolidated Standards of Reporting Trials
CRF: Case Report Form
HD: Hemodialysis
ICC: Intraclass Correlation Coefficient
IQR: Interquartile Range
MC-SCQ: Muscle Cramp Severity and Characteristics Questionnaire
RCT: Randomized Controlled Trial
RE-AIM: Reach, Effectiveness, Adoption, Implementation, Maintenance
SD: Standard Deviation
