# Supplementary material for: Effect of nurse-led intradialytic stretching exercises on muscle cramp burden among patients undergoing maintenance hemodialysis: a randomized controlled trial
Source: BMC Nurs. 2026 Mar 9;25:333. doi: 10.1186/s12912-026-04430-4 (PMC13063644; doi:10.1186/s12912-026-04430-4)

**Figure 1: CONSORT 2025 Flow Diagram**

Flow diagram of the progress through the phases of a randomised trial of two groups (that is, enrolment, intervention allocation, follow-up, and data analysis)


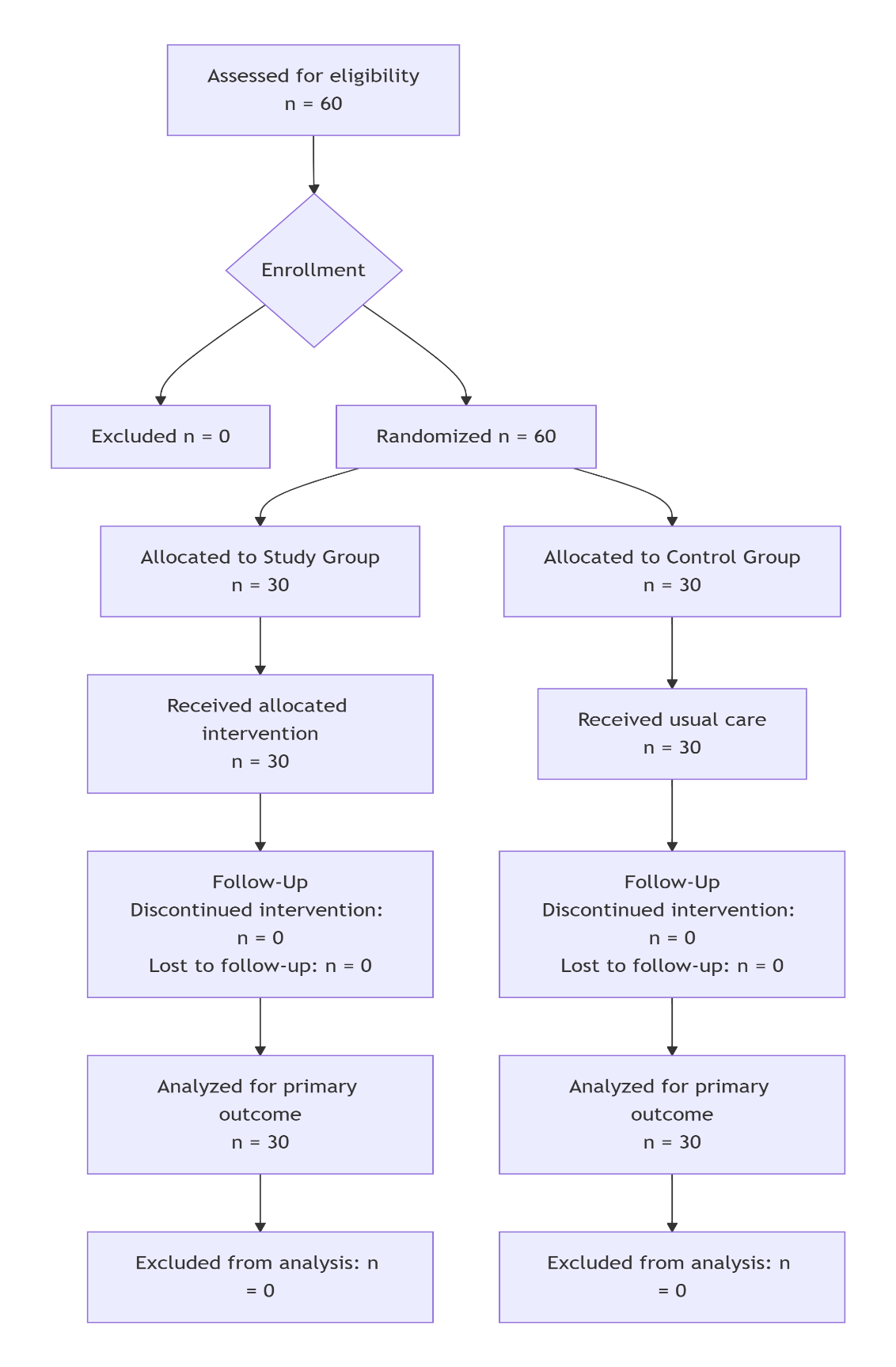

Supplement: Supplementary file 1 — Supplementary Material 1 [file 12912_2026_4430_MOESM1_ESM.docx]
