# Supplementary material for: Effect of nurse-led intradialytic stretching exercises on muscle cramp burden among patients undergoing maintenance hemodialysis: a randomized controlled trial
Source: BMC Nurs. 2026 Mar 9;25:333. doi: 10.1186/s12912-026-04430-4 (PMC13063644; doi:10.1186/s12912-026-04430-4)
Supplement: Supplementary file 2 — Supplementary Material 2 [file 12912_2026_4430_MOESM2_ESM.pdf]

Muscle Cramp Severity and Characteristics Questionnaire (MC-SCQ) – English Version

Developed based on the validated Arabic version for use in hemodialysis settings.

Introduction:
This questionnaire is designed to assess the severity and characteristics of muscle cramps experienced during hemodialysis sessions over a two-week period. Please answer each question based on your personal experience.

A. Frequency of Cramp Episodes (per hour of dialysis)
How often, on average, did cramps occur during each hour of dialysis?

- **0:** No cramps
- **1:** Less than 3 times per hour
- **2:** 3 times per hour
- **3:** More than 3 times per hour
  Score: _____

B. Average Duration of an Episode
How long did a typical cramp last?

- **0:** No cramps
- **1:** Less than 2 minutes
- **2:** 2–5 minutes
- **3:** More than 5 minutes
  Score: _____

C. Level of Pain During Cramps
How would you rate the pain caused by the cramps?

- **0:** No pain
- **1:** Mild (noticeable but does not interfere)
- **2:** Moderate (interferes with activity)
- **3:** Severe (debilitating, cannot continue activity)
  Score: _____

D. Leg Temperature Sensation During/After Cramp
Did you notice any change in the temperature or sensation of your leg during or after a cramp?

- **0:** No change / Neutral sensation
- **1:** Warm, Cold, or Clammy
  Score: _____

E. Overall Discomfort / Functional Interference
To what extent did the cramps cause discomfort or interfere with your ability to rest or engage in activity?

- **0:** No discomfort
- **1:** Perceptible (aware of it)
- **2:** Sensitive (somewhat bothersome)
- **3:** Painful (interferes with rest or activity)
- **4:** Unbearable (cannot tolerate)
  Score: _____

Scoring Summary

Total Score (A + B + C + D + E): _____ / 13

Cramp Intensity Category (Based on Total Score):

- **None:** 0
- **Mild:** 1–4
- **Moderate:** 5–8
- **Severe:** 9–13
